# Supplementary material for: Structure-based design of a Cortistatin analogue with immunomodulatory activity in models of inflammatory bowel disease
Source: Nat Commun. 2021 Mar 25;12:1869. doi: 10.1038/s41467-021-22076-5 (PMC7994712; doi:10.1038/s41467-021-22076-5)
Supplement: Supplementary file 1 — Supplementary Information [file 41467_2021_22076_MOESM1_ESM.pdf]

Supplementary information

Structure-based design of a Cortistatin analogue with immunomodulatory activity in models of inflammatory bowel disease

Álvaro Rol[a], Toni Todorovski[a, §], Pau Martin-Malpartida[a], Anna Escolà[a], Elena Gonzalez-Rey[b], Eric Aragón[a], Xavier Verdaguer[a,c], Mariona Vallès-Miret[d], Josep Farrera-Sinfreu[d], Eduard Puig[a], Jimena Fernández-Carneado[d], Berta Ponsati[d], Mario Delgado[b,*], Antoni Riera[a,c,*] and Maria J. Macias[a,e,*]

[a.] *Institute for Research in Biomedicine (IRB-Barcelona), The Barcelona Institute of Science and Technology, Baldiri i Reixac 10, Barcelona 08028, Spain.*

[b.] *Instituto de Parasitologia y Biomedicina López-Neyra, (IPBLN-CSIC) Avda. Conocimiento 17, Parque Tecnológico de la Salud, 18016 Armilla, Granada, Spain.*

[c.] *Departament de Química Inorgànica i Orgànica, Secció de Química Orgànica, Universitat de Barcelona, Martí i Franquès 1-11, 08028, Barcelona, Spain*

[d.] *BCN Peptides S.A. Pol.Ind. Els Vinyets-Els Fogars, Sector II. Ctra. Comarcal 244, Km. 22, 08777 Sant Quintí de Mediona, Barcelona 08777, Spain.*

[e.] *Institució Catalana de Recerca i Estudis Avançats (ICREA), Passeig Lluis Companys, 23, Barcelona 08010, Spain*

[§] *Present Address: Departament de Ciències Experimentals i de la Salut, Universitat Pompeu Fabra, Dr. Aiguader, 88 08003 Barcelona, Spain*

* *Authors to whom correspondence should be addressed: mdelgado@ipb.csic.es (M.D.), antoni.riera@irbbarcelona.org (A.R.), maria.macias@irbbarcelona.org (M.J.M.)*

Table of Contents

Supplementary Figures ... *3*
Supplementary Fig. 1 ... 3
Supplementary Fig. 2 ... 4
Supplementary Fig. 3 ... 5
Supplementary Fig. 4 ... 6
Supplementary Fig. 5 ... 7
Supplementary Fig. 6 ... 8
Supplementary Fig. 7 ... 10
Supplementary Tables ... *11*
Supplementary Table 1 ... 11
Supplementary Table 2 ... 11
Supplementary Table 3 ... 12
Supplementary Table 4 ... 12
Supplementary Notes ... *13*
Supplementary Note 1: NMR assignments, spectra and structure calculation statistics ... 13
Supplementary Note 2: Reverse phase – HPLC analysis of compounds ... 24
Supplementary Note 3: ESI-MS analysis of compounds ... 30
Supplementary References ... *33*

Supplementary Figures

a

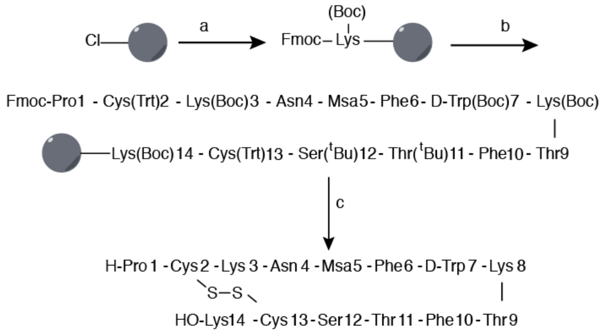

b

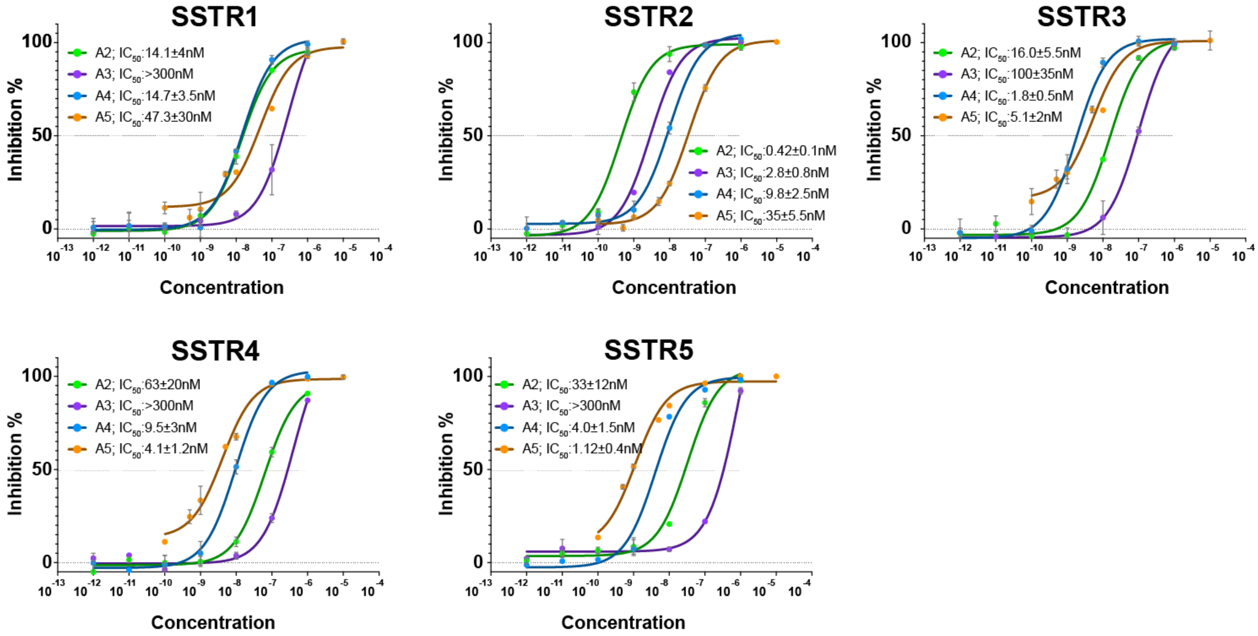

Supplementary Fig. 1.

a. Synthetic scheme. Synthesis of [L-Msa5_D-Trp7_L- Thr11]-CST14 **(**analogue **4).** All peptides have been prepared following a similar approach**.**

a) 1. Fmoc-L-Lys(Boc)-OH (3 equiv) or Fmoc-L-Cys(Trt)-OH (3 equiv), DIEA (3 equiv), 2. MeOH;
b) 1. Piperidine 20% DMF, 2. Fmoc-Aaa-OH (1.5–3 equiv, when the Msa amino acid was coupled, only 1.5 equivalents were used), DIPCDI (3 equiv), HOBt (3 equiv), DMF (x12), 3. Piperidine 20% DMF, 4. Fmoc-Pro-OH, DIPCDI, HOBt, DMF
c) 1. CH_2Cl_2/TFE/AcOH 2. I_2, 3. TFA/CH_2Cl_2/anisole/H2O. Boc=*tert*-butoxycarbonyl, DIEA=diisopropylethylamine, PCDI=diisopropylcarbodiimide, DMF= N,N'-dimethylformamide, Fmoc=N-(9- fluorenylmethoxycarbonyl), HOBT=1-hydroxybenzotriazole, TFA=trifluoroacetic acid, TFE=2,2,2-trifluoroethanol.

b. Binding curves and IC_{50} values for Cortistatin analogues with respect to Somatostatin receptors 1-5 (SSTR1-SSTR5), using a Radio-ligand Binding Assay (Supplementary Table 1). All analogues are studied at identical concentrations and compared to the same SST-labeled reference. Data is represented as mean values (n=2), error bars represent the 95% Confidence Interval of the curve fitting.

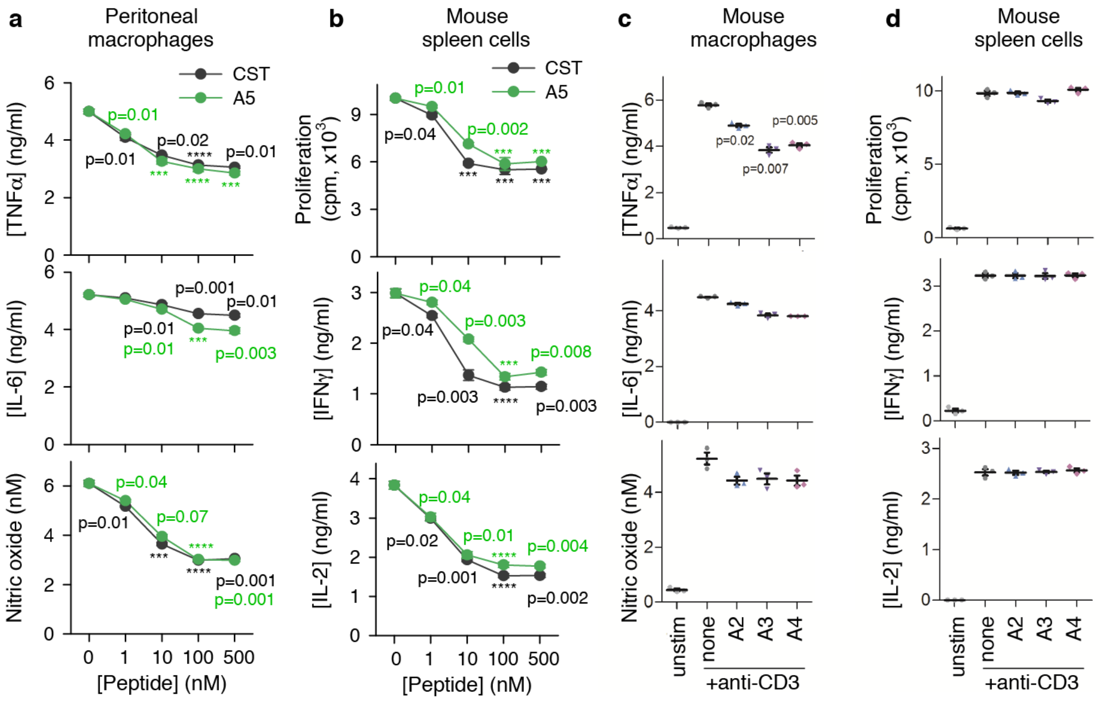

Supplementary Fig. 2. Cortistatin and analogue 5 deactivate macrophages and lymphocytes in a dose-response manner. a,b. Effects of various concentrations of Cortistatin (CST) and analogue 5 (A5) on the production of inflammatory cytokines and nitric oxide by LPS-activated mouse peritoneal macrophages (**a**) and on cell proliferation and production of Th1-type cytokines by anti-CD3-activated mouse spleen cells (**b**). **c,d.** Synthesized analogues **2**, **3** and **4** (A2, A3 and A4) did not significantly affect the inflammatory response of mouse Raw 264 macrophages (**c**) or T-cell activation of mouse spleen cells (**d**). Data are the mean ± SEM of three independent experiments (n=3), each performed as duplicates. Dots (in panels **c,d**) represent individual values of biologically independent cell cultures. Statistical significance between groups was assessed by paired, two-tailed Student's t-test. ****p*<0.001, *****p*<0.0001 vs. stimulated cells in the absence of peptides. Exact *p*-values are shown for *p*>0.001. Source data are provided as a Source Data file.

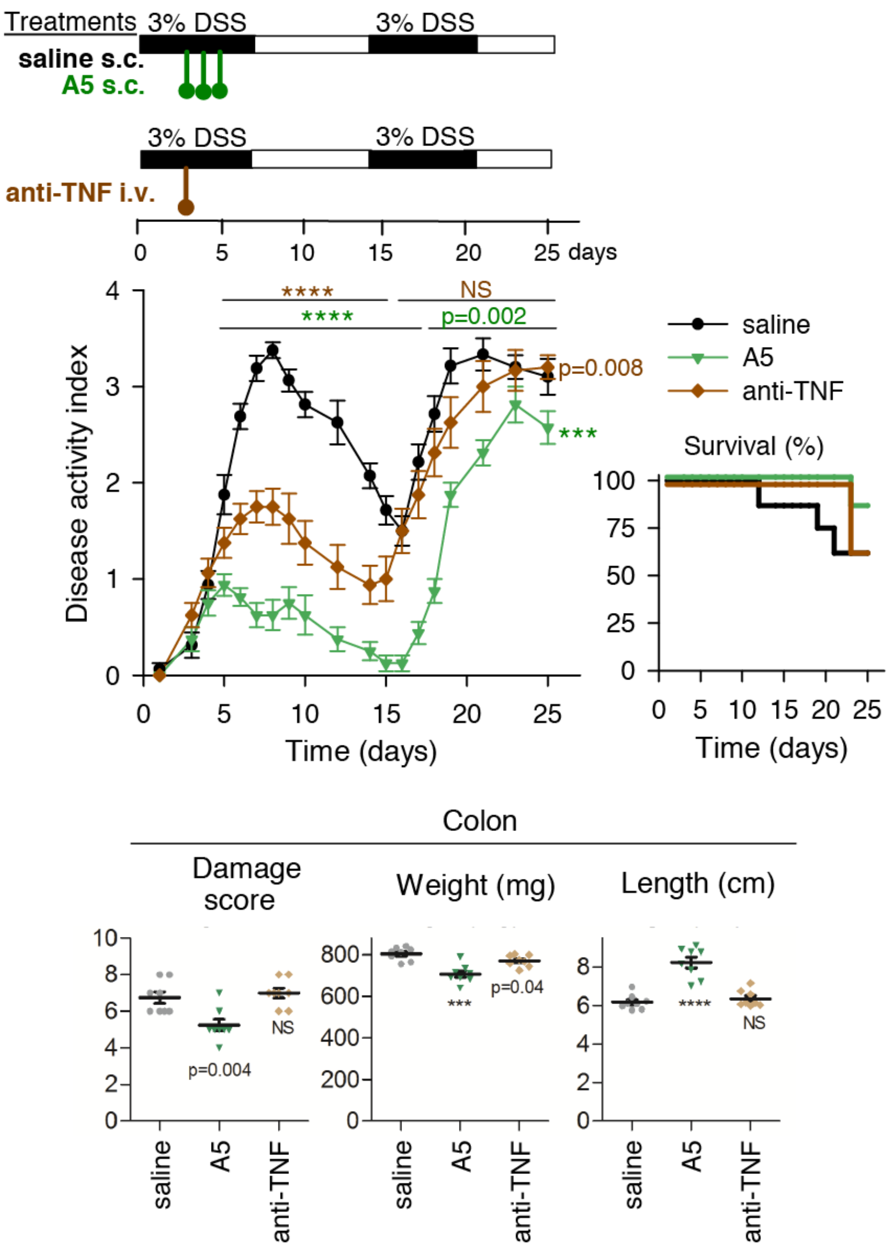

Supplementary Fig. 3. Tolerogenic effect of analogue 5 but not of anti-TNFα antibodies on DSS-induced ulcerative colitis. Comparative therapeutic effects in a model of relapsing-remitting colitis by treatments with analogue **5** (A5) and anti-mouse TNFα antibody only during the first cycle of DSS exposure (see scheme for experimental design). Clinical signs were evaluated by disease activity indexes (scoring body weight loss, stool consistency and presence of fecal blood), survival rate and macroscopic signs of colon inflammation (damage score, length and weight of colon). Animals injected with saline instead of A5 were used as untreated colitic mice. n=8 mice/group. Data are mean ± SEM with dots representing individual values of biologically independent animals. Statistical differences between groups were calculated using two-tailed non-parametric Mann-Whitney test (for disease activity index and colon damage score), unpaired two-tailed Student's t-test (for colon length and weight) and Kaplan-Meier test (for survival). ****p*<0.001; *****p*<0.0001 versus untreated DSS-colitic mice (saline). Exact *p*-values are shown for *p*>0.001. NS, not significant. Source data are provided as a Source Data file. Administrations: s.c., subcutaneous; i.v., intravenous.

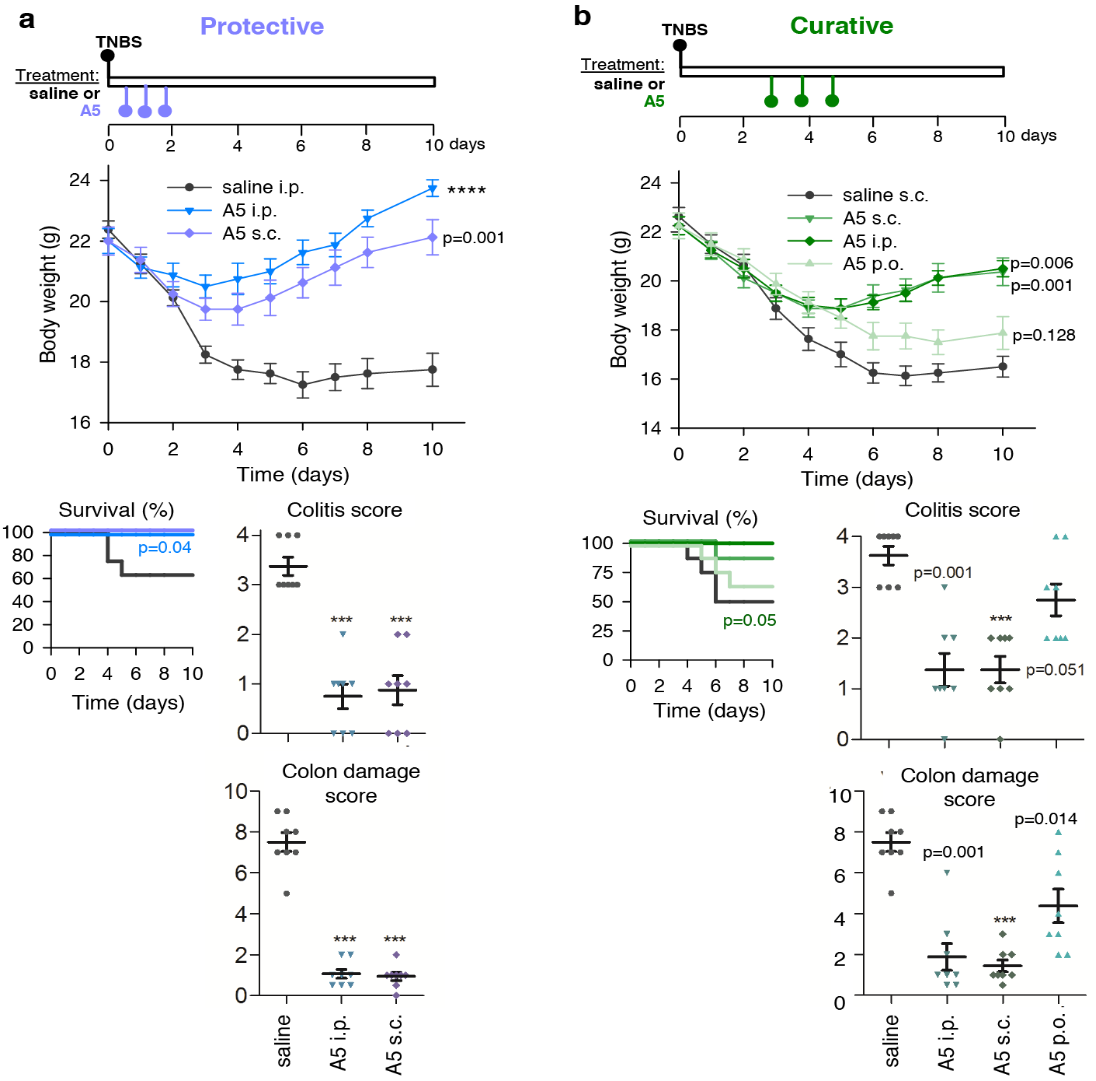

Supplementary Fig. 4. Protective and curative effects of analogue 5 on TNBS-induced acute colitis. Acute colitis was induced in mice by intrarectal TNBS and the animals were then treated with analogue **5** (A5) through different routes of administration following both protective (**a**) and curative (**b**) strategies as indicated in the schemes. Animals injected with saline instead of A5 were used as untreated colitic mice. Disease evolution and severity were monitored by survival and weight loss, colitis score and macroscopic colon damage score. n=8 mice/group. Data are mean ± SEM with dots representing individual values of biologically independent animals. Statistical differences between groups were calculated using two-tailed non-parametric Mann-Whitney test (for colon damage and colitis scores), unpaired two-tailed Student's t-test (for body weight) and Kaplan-Meier test (for survival). ***$p<0.001$; ****$p<0.0001$ versus untreated TNBS-colitic mice (saline). Exact p-values are shown for $p>0.001$. Source data are provided as a Source Data file. Administrations: s.c., subcutaneous; i.v., intravenous; p.o., oral.

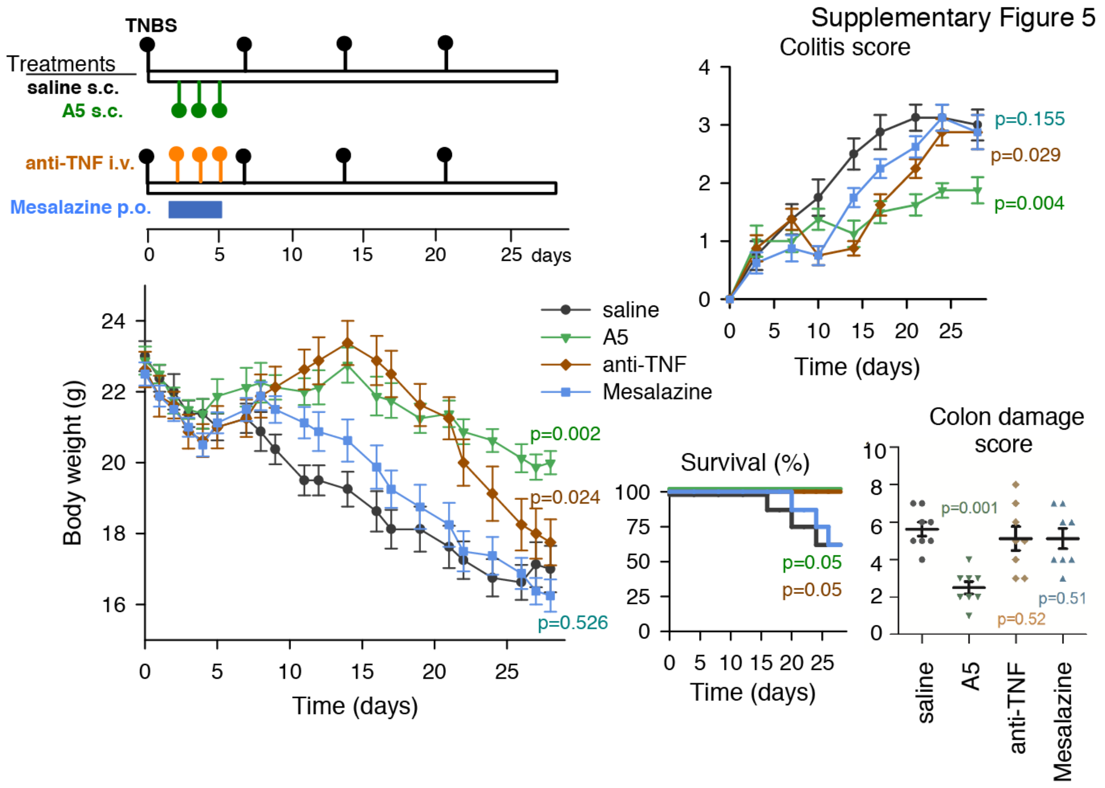

Supplementary Fig. 5. Tolerogenic effect of analogue 5 but not of treatments of reference on TNBS-induced chronic colitis. Chronic colitis was induced in mice by intrarectal injections of increasing doses of TNBS once a week and the animals were then treated with analogue **5** (A5), anti-mouse TNFα antibody or mesalazine only during the first week as indicated in the scheme. Animals injected with saline instead of A5 were used as untreated colitic mice. Disease evolution and severity were monitored by survival and weight loss, colitis score and macroscopic colon damage score. n=8 mice/group. Data are mean ± SEM with dots representing individual values of biologically independent animals. Statistical differences between untreated (saline) and treated groups were calculated using two-tailed non-parametric Mann-Whitney test (for colon damage and colitis scores), unpaired two-tailed Student's t-test (for body weight) and Kaplan-Meier test (for survival). Exact *p*-values are shown. Source data are provided as a Source Data file. Administrations: s.c., subcutaneous; i.v., intravenous; p.o., oral.

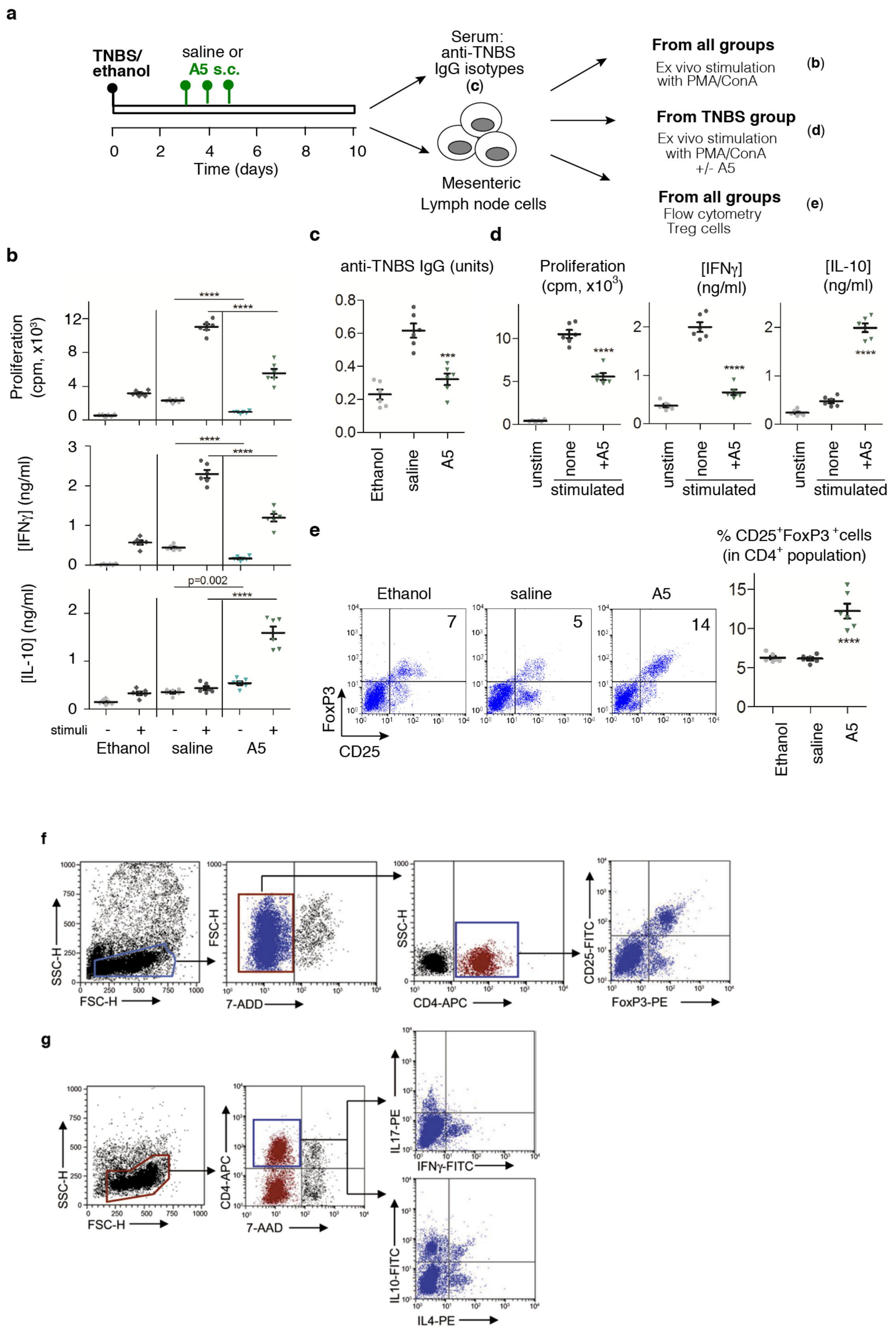

Supplementary Fig. 6. Treatment with analogue 5 regulates Th1- and Th17-induced inflammatory responses and triggers regulatory T cells in TNBS-induced colitis. a. Mice (n=6 mice/group) with TNBS-induced acute colitis were treated with saline or analogue **5** (A5) as indicated in the scheme. Mice injected with 50% ethanol were used as basal controls. **b.** Cell proliferation and cytokine production by mesenteric lymph node (MLN) cells isolated from all experimental groups in basal conditions (stimuli: -)

or in response to T-cell stimulation (PMA+ConA, stimuli: +). **c.** Levels of IgG autoantibodies for TNBS in sera. **d.** Cell proliferation and cytokine production by MLN cells isolated from untreated colitic mice in basal conditions (unstimulated) or in response to ex vivo stimulation (PMA+ConA) in the absence (none) or presence of A5. **e.** Flow cytometry analysis of regulatory T cells in MLNs isolated from all experimental groups (results show the percentage of $CD25^{+}FoxP3^{+}$ cells in gated $CD4^{+}$ T cell population). Data are mean ± SEM with dots representing individual values of biologically independent animals. Statistical differences between groups were calculated using unpaired two-tailed Student's t-test. ****p*<0.001; *****p*<0.0001 versus untreated TNBS-induced colitic mice (saline) or versus untreated stimulated-MLN cells (none, panel **d**). Exact *p*-values are shown for *p*>0.001. Source data are provided as a Source Data file. **f.** Gating strategy to identify CD25+FoxP3+ regulatory T cells (Treg) in the CD4+ population of mesenteric lymph nodes isolated from colitic mice shown in Fig. 6c and Supplementary Fig. 6e. **g.** Gating strategy to identify the percentage of CD4+ lymphocytes producing Th1, Th17 and Treg cytokines in activated mesenteric lymph node cell cultures shown in Fig. 6b. Staining with 7-aminoactinomycin D (7-AAD) was used to exclude dead cells in all analyses.

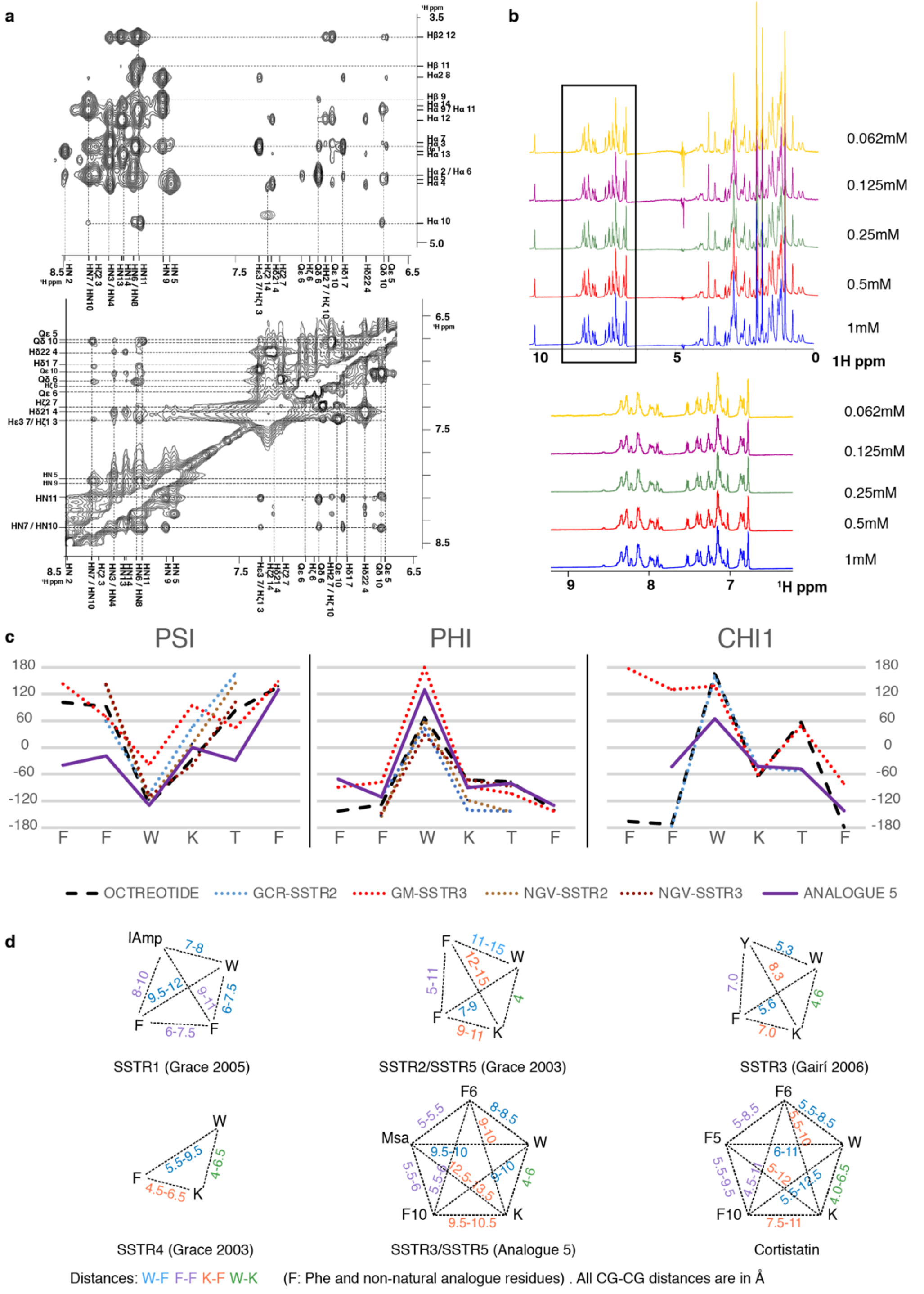

Supplementary Fig. 7. Structural properties of analogue 5, (A5). a. Amide/proton alpha (above) and amide/aromatic (below) regions of the analogue **5** (^{1}H-NOESY experiment), with peak assignments displayed. **b.** Comparison of the chemical shifts (full 1D spectrum of analogue **5**) are shown as a dilution series of spectra recorded at 1, 0.5, 0.25, 0.125 and 0.062 mM of a peptide solution in 90%H_2O/10%D_2O. No differences are observed between the different datasets with the exception of the signal to noise ratio that is affected by the dilution. **c.** Comparison of Phi, Psi and Chi1 values of A**5** to Octreotide and four SST analogues described in the literature. These SST analogues were selected for the comparison, due to their specific preferences towards different SSTRs. **d.** Pharmacophores described in the literature for SST analogues and for Cortistatin and A**5** described in this work**.** The pharmacophores are depicted as distances between key residues (gamma Carbons). Distances are represented as ranges to include the distance dispersion in the ensemble of conformations.

Supplementary Tables

Supplementary Table 1. Pharmacological profile of analogues 2-5, Cortistatin and Somatostatin

	SSTR1 (nM)	SSTR2 (nM)	SSTR3 (nM)	SSTR4 (nM)	SSTR5 (nM)
**Somatostatin**	0.1-2.26*<br>0.2±0.1	0.2-1.3*<br>0.4±0.2	0.3-1.6*<br>0.3±0.1	0.3-1.8*<br>0.5±0.2	0.2-0.9*<br>0.2±0.06
**Cortistatin**	1.7-5.0*	0.09-1.8*	0.3-3.8*	0.2-18.2*	0.3-1.9*
**Analogue 2 (A2)**	14.1±4	**0.42±0.1**	16.0±5.5	63±20	33±12
**Analogue 3 (A3)**	>300	2.8±0.8	100±35	>300	>300
**Analogue 4 (A4)**	14.7±3.5	9.8±2.5	**1.8±0.5**	**9.5±3**	4.0±1.5
**Analogue 5 (A5)**	47.3±30	35±5.5	5.1±2	**4.1±1.2**	**1.12±0.4**

Binding curves and IC_{50} values for Cortistatin analogues with respect to Somatostatin receptors 1-5 (SSTR1-SSTR5), using a Radio-ligand Binding Assay (Supplementary Figure 1b). Cells were incubated in HEPES buffer pH 7.4 with the new analogues of Cortistatin at 10 different concentrations in a range of 0.1 nM to 10 µM for 2-4 h and ^{125}I-Tyr11 SST14 was used as radio-ligand and SST14 as cold ligand. Membranes were filtered and washed 3 times and the filters were counted to determine [^{125}I]-SST-14 specifically bound (performed at Eurofins Panlabs, Inc.). The radioactivity obtained in the absence of SST14 is considered as total binding and that obtained in the presence of 1 µM of SST14 is considered as nonspecific binding. Specific binding was considered as the difference between total and nonspecific. Biochemical assay results are presented as the percent inhibition of specific binding (% inhibition) as a mean of duplicates (n=2), and error bars represent the 95% Confidence Interval of the curve fitting. The reliability of the assay was assessed by using SST-14 reference standards that were run as an integral part of each binding assay with the five (SSTR1-SSTR5). IC_{50} values were determined by a non-linear, least squares regression analysis using Prism 9 (GraphPad Software, USA), using the Dose-response inhibition (three-parameters) model.

*Data from [1]

Supplementary Table 2. Viability and apoptosis of spleen cells and macrophages activated in the absence of presence of analogue 5 (A5).

	Viability (%)	Apoptosis (%)
**Macrophages: unstimulated**	97±1.4	3.2±0.6
**LPS**	94±1.1	9.2±1.2
**LPS+A5**	93±1.0	9.0±0.4
**Spleen cells: unstimulated**	98±0.6	3.4±0.9
**anti-CD3**	92±1.2	10.6±1.0
**anti-CD3+A5**	92±0.4	9.2±0.9

Mouse peritoneal macrophages were cultured in medium (unstimulated) or stimulated with LPS (1 μg/ml) in the absence or presence of Analogue 5 (100 nM). Mouse spleen cells were cultured in medium alone (unstimulated) or stimulated with anti-CD3 antibodies (2 μg/ml) in the absence or presence of analogue **5** (100 nM). After 24 hours (macrophages) or 48 hours (spleen cells) of culture, cell viability and apoptosis were determined. The percentage of cell viability relative to the initiation of cell culture was determined using AlamarBlue assay (Thermo Fisher Scientific). The percentage of apoptotic cells was determined by flow cytometric analysis of Annexin $V^{positive}$ 7-amino-actinomycin $D^{negative}$ cell population (R&D Systems). Data are the mean ± SEM of 5 independent cultures performed in duplicates. No statistical significant differences were observed between groups using an unpaired, two-tailed Student's t-test (p>0.05).

Supplementary Table 3. Serum cytokines in TNBS-induced acute colitis.

**Treatments**	**TNFα (pg/ml)**	**IL6 (pg/ml)**	**IL17 (pg/ml)**
**Ethanol**	200±14	255±18	97±31
**TNBS+saline**	1617±50	1603±71	915±49
**TNBS+A5**	707±43 ****	735±51 ****	268±18 ****
**TNBS+anti-TNFα**	421±37 ****	932±40 ****	358±24 ****
**TNBS+Mesalazine**	782±60 ****	773±46 ****	233±15 ****

Treatments were administered in a curative regime as described in Figure 4 and sera were collected 10 days after TNBS infusion. n=8 mice/group. Data are mean ± SEM. Statistical differences between groups were calculated using unpaired two-tailed Student's t-test. ****p<0.0001 versus TNBS+saline group.

Supplementary Table 4. Oligos used in the determination of gene expression by real-time PCR

	forward	reverse
T-bet	5′-CCAGGGAACCGCTTATATGT-3	5′-CTGGGTCACATTGTTGGAAG-3
Foxp3	5′-GGCCCTTCTCCAGGACAGA-3′;	5′-GCTGATCATGGCTGGGTTGT-3
RORγt	5′-CCACTGCATTCCCAGTTTCT-3′	5-CGTAGAAGGTCCTCCAGTCG-3′
β-actin	5′-TTCCAGCGTTCCTTCTTGGGTAT-3′	5′-GTTGGCATAGAGGTGTTTACGG-3′

Total RNA was isolated from colon sections obtained at day 10 after TNBS injection following the manufacturer's protocol (Tripure, Roche). Precipitated RNA was treated with DNase 1 (Sigma) before reverse transcription (RevertAid First Strand cDNA Synthesis Kit, ThermoFisher Scientific). SYBER green quantitative PCR (SensiFast Sybr No-Rox mix, Bioline) was performed as described in the Methods Section.

Supplementary Notes

Supplementary Note 1: NMR assignments, spectra and structure calculation statistics.

CST14, 1: Cortistatin**1** was synthesized following the general procedure from 0.25 g of 2-Cl-Trt resin (1.60 mmol/g) and using Boc-Pro-OH as N-terminal amino acid. ESI-MS: M theoretical mass 1777 g/mol, experimental mass 1778±1 (m/z): [M+2H]+/2=889.3, [M+3H]+/3=593.1.

	HN	Hα	Hβ	Hγ	Hδ	Hε	Hζ	Hη
**1 Pro**	7.95	4.15	2.18 (β2)<br>1.77 (β3)	1.76	3.14	-	-	-
**2 Cys**	8.67	4.43	2.90 (β2)<br>2.77 (β3)	-	-	-	-	-
**3 Lys**	8.56	4.04	1.45	1.12 (γ2)<br>1.06 (γ3)	1.39	2.68	-	-
**4 Asn**	8.11	4.38	2.34	-	7.29 (δ21)<br>6.66 (δ22)	-	-	-
**5 Phe**	8.03	4.14	2.53 (β2)<br>2.47 (β3)	-	6.80	7.03	6.96	-
**6 Phe**	7.95	4.28	2.76 (β2)<br>2.69 (β3)	-	6.89	7.05	-	-
**7 Trp**	7.56	4.34	2.99	-	6.90	10.01 (ε1)<br>7.23 (ε3)	7.17 (ζ2)<br>6.88 (ζ3)	6.96
**8 Lys**	7.74	3.80	1.44 (β2)<br>1.34 (β3)	0.80	1.28	2.60	-	-
**9 Thr**	7.53	4.00	3.94	0.82	-	-	-	-
**10 Phe**	7.99	4.36	2.89 (β2)<br>2.75 (β3)	-	6.95	7.05	-	-
**11 Ser**	8.03	4.23	3.53	-	-	-	-	-
**12 Ser**	8.02	4.26	3.63 (β2)<br>3.60 (β3)	-	-	-	-	-
**13 Cys**	8.23	4.47	2.94 (β2)<br>2.74 (β3)	-	-	-	-	-
**14 Lys**	7.94	3.94	1.55 (β2)<br>1.44 (β3)	1.10	1.36	2.66	-	-

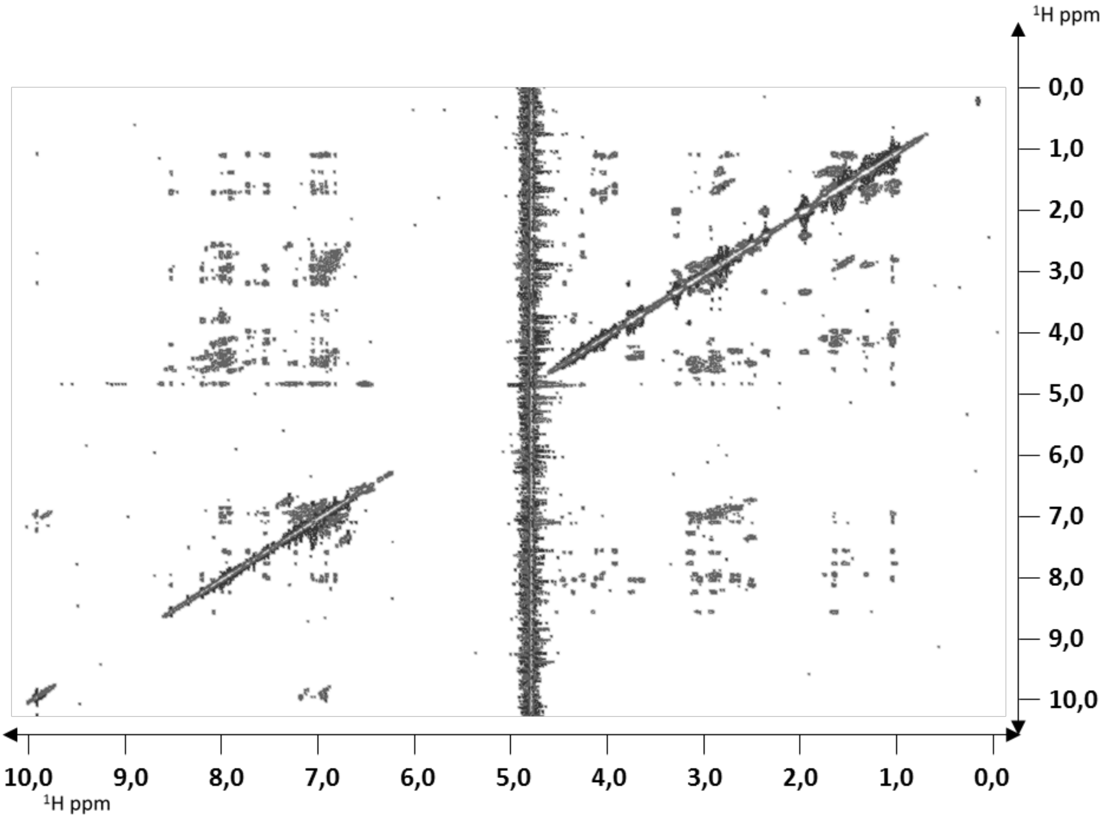

NOESY 350 ms for analogue **1**

[L-Msa6_D-Trp7_L-Thr11]-CST14, 2: Cortistatin Analogue **2** was synthesized following the general procedure from 0.25 g of 2-Cl-Trt resin (1.60 mmol/g) and using Boc-Pro-OH as N-terminal amino acid, affording 0.56 g of crude. ESI-MS: Theoretical mass 1777 g/mol, experimental 1778±1 (m/z): [M+2H]+/2=889.2, [M+3H]+/3=593.1.

	HN	Hα	Hβ	Hγ	Hδ	Hε	Hζ	Hη
**1 Pro**	-	4.16	2.21 (β2)<br>2.19 (β3)	1.81 (γ2)<br>1.77 (γ3)	3.17 (δ2)<br>3.14 (δ3)	-	-	-
**2 Cys**	8.71	4.45	2.88 (β2)<br>2.76 (β3)	-	-	-	-	-
**3 Lys**	8.44	4.35	1.36	1.07 (γ2)<br>0.97 (γ3)	1.23 (δ2)<br>1.13 (δ3)	2.49	-	-
**4 Asn**	8.30	4.56	2.44 (β2)<br>2.35 (β3)	-	7.33 (δ21)<br>6.75 (δ22)	-	-	-
**5 Phe**	8.03	4.14	2.53 (β2)<br>2.47 (β3)	-	6.70	6.94	6.89	-
**6 Msa**	8.14	4.31	2.79	-	2.03*	6.68	-	1.74
**7 DTrp**	8.12	4.24	2.82 (β2)<br>2.75 (β3)	-	6.85	10.01 (ε1)<br>7.33 (ε3)	7.25 (ζ2)<br>6.91 (ζ3)	7.00
**8 Lys**	8.10	3.90	1.35 (β2)<br>0.99 (β3)	0.29 (γ2)<br>0.10 (γ3)	1.28	2.45 (ε2)<br>2.37 (ε3)	-	-
**9 Thr**	7.79	4.16	3.94	0.89	-	-	-	-
**10 Phe**	8.11	4.82	2.57 (β2)<br>2.51 (β3)	-	6.83	7.04	6.99	-
**11 Thr**	8.22	4.22	3.98	0.93	-	-	-	-
**12 Ser**	8.22	4.30	3.67	-	-	-	-	-
**13 Cys**	8.26	4.38	3.66 (β2)<br>2.79 (β3)	-	-	-	-	-
**14 Lys**	7.91	3.91	1.56 (β2)<br>1.45 (β3)	1.13	1.41	2.72	-	-

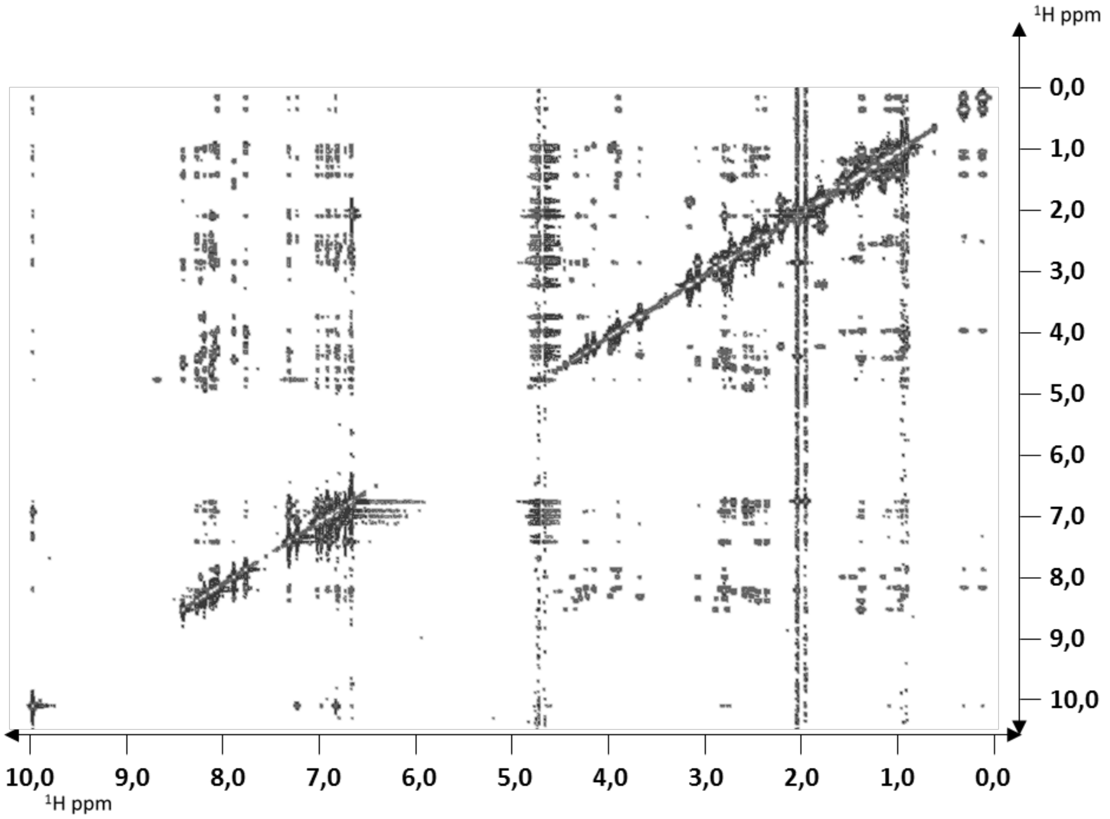

NOESY 350 ms (analogue **2**

DistanceRestricions:

Intraresidual: 0
Sequential: 92
Medium-range (1<dist≤4): 52
Long-range (dist>4): 56
Total: 200
Dihedralanglerestrictions: 24
Statisticsfor 20 beststructures

**Energies(kcal/mol):**	**RMSD*:**
Total energy: 26.09 ± 20.49	Bonds (Å): 1.000 x10^{-2} ± 4.043x10^{-4}
Van der Waals: 106.8 ± 8.184	Angles (°): 1.806 ± 0.04618
Electrostatic: -495.3 ± 30.44	Impropers (°): 2.617 ± 0.2759
Bonds: 25.56 ± 2.125	Dihedrals (°): 43.14 ± 0.1599
Angles: 229.0 ± 12.07	NOEs: 1.196 x10^{-2} ± 4.760 x10^{-4}

Structural Statistics for the 20 Lowest Energy Structures of [L-Msa6_D-Trp7_L-Thr11]-CST14 (**2**). *R.m.s deviation between the ensemble of the 20 lowest energy structures and the lowest energy structure.

Octanoyl-[L-Msa6_D-Trp7_L-Thr11]-CST14 3: CortistatinAnalogue **3** was synthesized following the general procedure from 0.25 g of 2-Cl-Trt resin (1.60 mmol/g) and using Fmoc-Pro-OH as N-terminal amino acid. Octanoic acid was introduced with 5 eq. of acid, 5 eq. of HOBT and 5 eq. of DIPCDI, yielding 0.50 g of crude. ESI-MS: theoretical mass 1903 g/mol, experimental mass 1905±1 (m/z): [M+2H]+/2=952.4, [M+3H]+/2= 635.2.

	HN	Hα	Hβ	Hγ	Hδ	Hε	Hζ	Hη
**1 Pro**	-	4.12	2.01 (β2)<br>1.72 (β3)	1.67 (γ2)<br>1.65 (γ3)	3.42 (δ2)<br>3.38 (δ3)	-	-	-
**2 Cys**	8.13	4.38	2.92 (β2)<br>2.73 (β3)	-	-	-	-	-
**3 Lys**	8.28	4.34	1.39 (β2)<br>1.07 (β3)	1.34 (γ2)<br>1.20 (γ3)	0.97	2.49	-	-
**4 Asn**	8.29	4.56	2.39	-	7.33 (δ21)<br>6.76 (δ22)	-	-	-
**5 Phe**	8.13	4.49	2.71 (β2)<br>2.59 (β3)	-	6.70	6.93	6.89	-
**6 Msa**	8.11	4.32	2.80	-	2.03*	6.68	-	1.95
**7 DTrp**	8.08	4.25	2.81 (β2)<br>2.75 (β3)	-	6.84	10.01 (ε1)<br>7.33 (ε3)	7.25 (ζ2)<br>6.92 (ζ3)	7.00
**8 Lys**	8.08	3.90	1.34 (β2)<br>1.00 (β3)	0.31 (γ2)<br>0.12 (γ3)	1.08	2.46 (ε2)<br>2.38 (ε3)	-	-
**9 Thr**	7.78	4.14	3.94	0.87	-	-	-	-
**10 Phe**	8.03	4.80	2.56 (β2)<br>2.49 (β3)	-	6.83	7.04	7.00	-
**11 Thr**	8.19	4.20	3.98	0.93	-	-	-	-
**12 Ser**	8.18	4.31	3.68	-	-	-	-	-
**13 Cys**	8.15	4.29	3.08 (β2)<br>2.74 (β3)	-	-	-	-	-
**14 Lys**	7.88	3.91	1.56 (β2)<br>1.45 (β3)	1.13	1.41	2.73	-	-

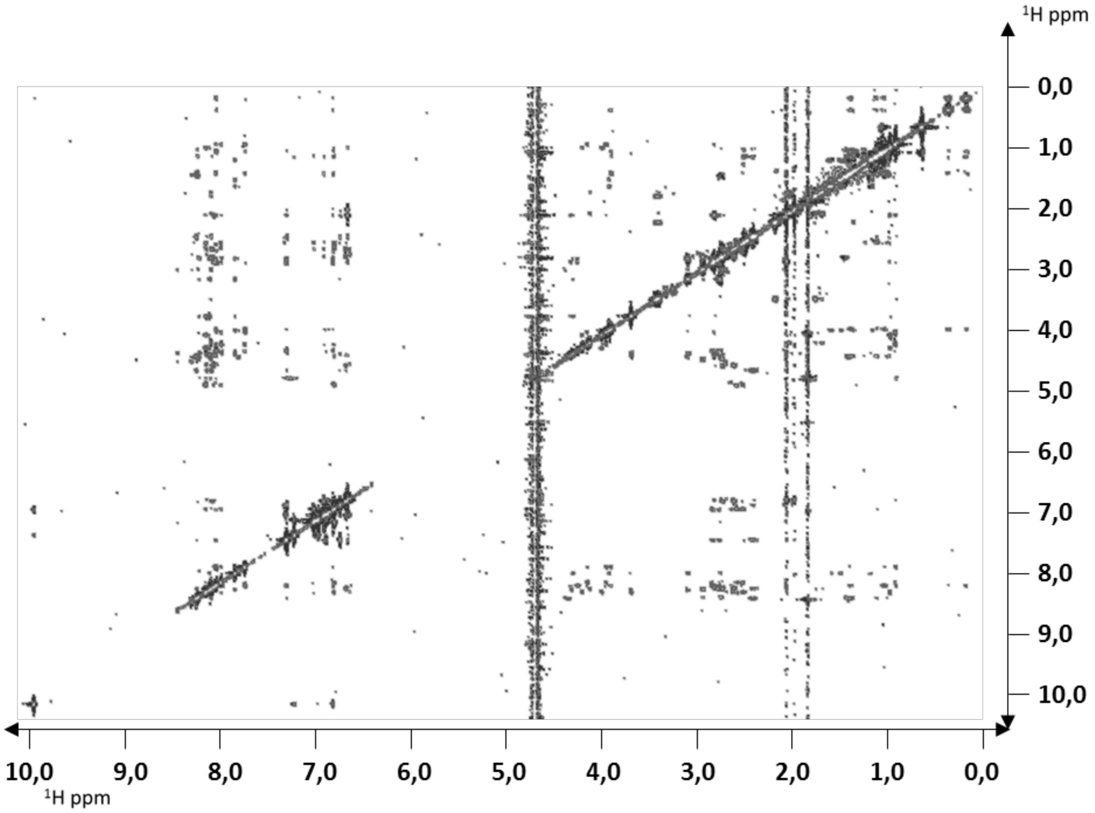

NOESY 350 ms (analogue **3)**

DistanceRestricions:

Intraresidual: 0

Sequential: 65

Medium-range (1<dist≤4): 21

Long-range (dist>4): 21

Total: 107

Dihedralanglerestrictions: 0

Statisticsfor 20 beststructures

**Energies (kcal/mol):**	**RMSD*:**
Total energy: -273.3 ± 18.53	Bonds (Å): 3.955 x10^{-3} ± 2,771x10^{-4}
Van der Waals: -22.48 ± 10.92	Angles (°): 1.216 ± 0.03701
Electrostatic: -452.4 ± 33.94	Impropers (°): 1.430 ± 0.344
Bonds: 4.008 ± 0.5717	Dihedrals (°): 42.03 ± 1.021
Angles: 103.9 ± 6.411	NOEs: 6.337 x10^{-3} ± 1,179 x10^{-3}

Structural Statistics for the 20 Lowest Energy Structures ofoctanoyl-[L-Msa6_D-Trp7_L-Thr11]-CST14 (**3**). *R.m.s deviation between the ensemble of the 20 lowest energy structures and the lowest energy structure.

[L-Msa5_D-Trp7_L-Thr11]-CST14, 4: Cortistatin Analogue **4** was synthesized following the general procedure from 0.25 g of 2-Cl-Trt resin (1.60 mmol/g) and using Boc-Pro-OH as N-terminal amino acid, affording 0.53 g of crude. ESI-MS: Theoretical mass 1777 g/mol, experimental 1778 ±1 (m/z): [M+2H]+/2=889 [M+3H]+/2= 593.

	HN	Hα	Hβ	Hγ	Hδ	Hε	Hζ	Hη
**1 Pro**	7.74	4.16	2.21	1.81 (γ2)<br>1.78 (γ3)	3.17 (δ2)<br>3.14 (δ3)	-	-	-
**2 Cys**	8.00	4.41	2.92 (β2)<br>2.75 (β3)	-	-	-	-	-
**3 Lys**	8.42	4.30	1.45	1.16 (γ2)<br>1.05 (γ3)	1.35	2.64	-	-
**4 Asn**	8.27	4.51	2.27	-	7.33 (δ21)<br>6.74 (δ22)	-	-	-
**5 Msa**	7.82	4.56	2.77 (β2)<br>2.68 (β3)	-	1.93*	6.65	-	1.84
**6 Phe**	8.16	4.46	2.79 (β2)<br>2.74 (β3)	-	7.03	7.12	7.07	-
**7 DTrp**	8.42	4.29	2.84	-	6.88	10.00 (ε1)<br>7.36 (ε3)	7.24 (ζ2)<br>6.90 (ζ3)	6.99
**8 Lys**	8.11	3.87	1.37 (β2)<br>0.97 (β3)	0.25 (γ2)<br>0.06 (γ3)	1.06	2.43 (ε2)<br>2.34 (ε3)	-	-
**9 Thr**	7.97	4.05	4.03	0.90	-	-	-	-
**10 Phe**	8.46	4.76	2.64	-	6.60	6.91	6.97	-
**11 Thr**	8.08	4.06	3.76	0.85	-	-	-	-
**12 Ser**	8.11	4.08	3.62	-	-	-	-	-
**13 Cys**	8.22	4.36	3.02 (β2)<br>2.74 (β3)	-	-	-	-	-
**14 Lys**	7.86	3.91	1.55 (β2)<br>1.44 (β3)	1.12	1.40	2.71	-	-

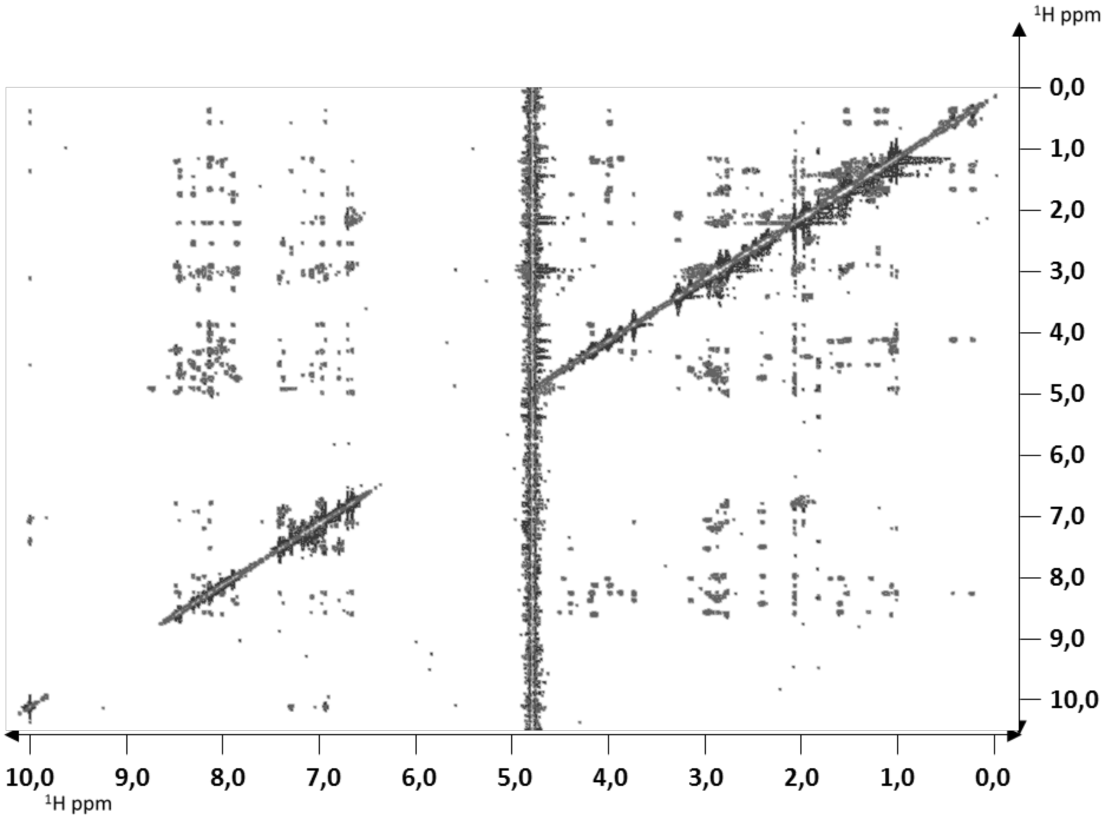

NOESY 350 ms (analogue **4)**

DistanceRestricions:

Intraresidual: 0

Sequential: 78

Medium-range (1<dist≤4): 37

Long-range (dist>4): 50

Total: 165

Dihedralanglerestrictions: 20

Statisticsfor 20 beststructures

**Energies (kcal/mol):**	**RMSD*:**
Total energy: -77.74 ± 18.24	Bonds (Å): 6.250 $x10^{-3}$ ± $2.751x10^{-4}$
Van der Waals: 1.073 ± 7.874	Angles (°): 1.347 ± 0.03784
Electrostatic: -404.2 ± 17.43	Impropers (°): 4.179 ± 0.2102
Bonds: 10.06 ± 0.8831	Dihedrals (°): 43.40 ± 0.5269
Angles: 128.3 ± 7.282	NOEs: 7.625 $x10^{-3}$ ± 5.385 $x10^{-4}$

Structural Statistics for the 20 Lowest Energy Structures of [L-Msa5_D-Trp7_L-Thr11]-CST14 (**4**). *R.m.s deviation between the ensemble of the 20 lowest energy structures and the lowest energy structure.

Octanoyl-[L-Msa5_D-Trp7_L-Thr11]-CST14, 5: Cortistatin Analogue **5** was synthesized following the general procedure from 0.25 g of 2-Cl-Trt resin (1.60 mmol/g) and using Fmoc-Pro-OH as N-terminal amino acid, and octanoic acid was introduced with 5 eq. of acid, 5 eq. of HOBT and 5 eq. of DIPCDI, yielding 0.55 g of crude. ESI-MS: Theoretical mass 1903 g/mol, experimental 1905±1 (m/z): [M+2H]+/2=952.4, [M+3H]+/2= 635.2.

	HN	Hα	Hβ	Hγ	Hδ	Hε	Hζ	Hη
**1 Pro**	-	4.12	2.13 (β2)<br>1.72 (β3)	1.66	3.38	-	-	-
**2 Cys**	8.15	4.37	2.88 (β2)<br>2.78 (β3)	-	-	-	-	-
**3 Lys**	8.23	4.26	1.44	1.14 (γ2)<br>1.04 (γ3)	1.30	2.61	7.31	-
**4 Asn**	8.23	4.56	2.31	-	7.28 (δ21)<br>6.73 (δ22)	-	-	-
**5 Msa**	7.88	4.55	2.72	-	1.90*	6.61	-	1.82
**6 Phe**	8.16	4.46	2.75	-	7.01	7.10	7.06	-
**7 DTrp**	8.35	4.27	2.83	-	6.86	9.97 (ε1)<br>7.35 (ε3)	7.23 (ζ2)<br>6.91 (ζ3)	6.98
**8 Lys**	8.08	3.87	1.35 (β2)<br>0.94 (β3)	0.28 (γ2)<br>0.10 (γ3)	1.07	2.45 (ε2)<br>2.35 (ε3)	7.23	-
**9 Thr**	7.92	4.06	4.00	0.89	-	-	-	-
**10 Phe**	8.36	4.29	2.62	-	6.63	6.93	6.67	-
**11 Thr**	8.05	4.06	3.80	0.85	-	-	-	-
**12 Ser**	8.07	4.11	3.63	-	-	-	-	-
**13 Cys**	8.16	4.33	3.01 (β2)<br>2.72 (β3)	-	-	-	-	-
**14 Lys**	8.15	4.03	1.49	1.41 (γ2)<br>1.16 (γ3)	1.63	2.72	-	-

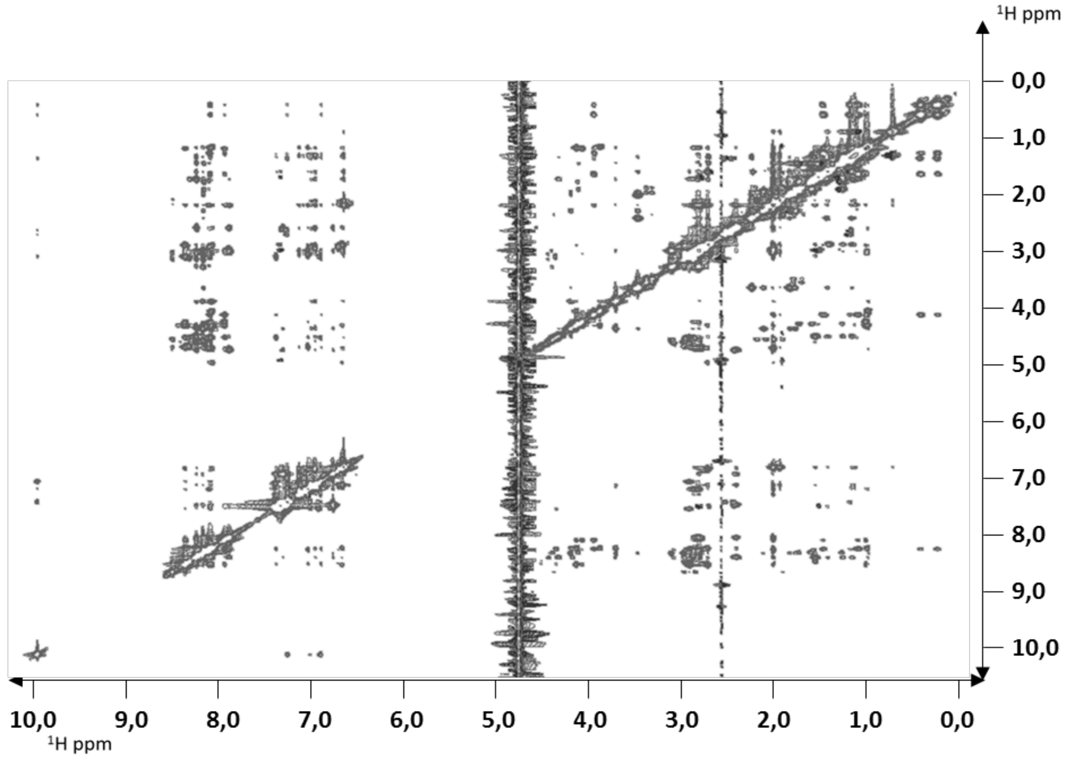

NOESY 350 ms (analogue **5**)

DistanceRestricions:

Intraresidual: 0

Sequential: 93

Medium-range (1<dist≤4): 31

Long-range (dist>4): 49

Total: 173

Dihedral angle restrictions: 24

Statistics for 20 best structures

**Energies (kcal/mol):**	**RMSD*:**
Total energy: -313.9 +/- 15.1	Bonds (Å): $3.8 \times 10^{-3} \pm 1.4 \times 10^{-4}$
Van der Waals: -51.94 +/- 6.6	Angles (º): 1.05 ± 0.01
Electrostatic: -443.4 +/- 21.7	Impropers (º): 1.44 ± 0.2
Bonds: 4.024 +/- 0.31	Dihedrals (º): 39.31 ± 0.2
Angles: 85.96 +/- 1.78	NOEs: $6.0 \times 10^{-3} \pm 2.6 \times 10^{-4}$

Structural Statistics for the 20 Lowest Energy Structures ofoctanoyl-[L-Msa5_D-Trp7_L-Thr11]-CST14 (**5**). *R.m.s deviation between the ensemble of the 20 lowest energy structures and the lowest energy structure. These structures are deposited at the PDB (6Y1Q). The validation analysis is provided. Small differences with respect to ideal values are observed due to the presence of non-natural amino acids.

Octanoyl-[L-Msa5_D-Trp7_L-Thr11]-CST13, 6: Cortistatin Analogue **6** was synthesized following the general procedure from 0.25 g of 2-Cl-Trt resin (1.60 mmol/g) and using Fmoc-Pro-OH as N-terminal amino acid, and octanoic acid was introduced with 5 eq. of acid, 5 eq. of HOBT and 5 eq. of DIPCDI, affording 0.53 g of crude. ESI-MS: M Theoretical mass 1775 g/mol, experimental 1777±1 (m/z): [M+2H]+/2=888, [M+3H]+/3= 592.

	HN	Hα	Hβ	Hγ	Hδ	Hε	Hζ	Hη
**1 Pro**	-	4.11	2.01	1.73 (γ2)<br>1.66 (γ3)	3.40	-	-	-
**2 Cys**	8.40	4.29	2.85	-	-	-	-	-
**3 Lys**	8.25	4.30	1.43	1.15 (γ2)<br>1.02 (γ3)	1.29 (δ2)<br>1.22 (δ3)	2.58	-	-
**4 Asn**	8.24	4.55	2.30	-	7.27 (δ21)<br>6.73 (δ22)	-	-	-
**5 Msa**	8.18	4.47	2.79 (β2)<br>2.69 (β3)	-	1.91*	6.62	-	1.80
**6 Phe**	8.18	4.47	2.79 (β2)<br>2.73 (β3)	-	7.03	7.11	7.07	-
**7 DTrp**	7.86	4.16	2.84	-	6.88	9.99 (ε1)<br>7.36 (ε3)	7.24 (ζ2)<br>6.90 (ζ3)	6.99
**8 Lys**	7.85	2.91	1.25	1.03	1.34	1.98	-	-
**9 Thr**	7.93	4.09	4.00	0.89	-	-	-	-
**10 Phe**	8.33	4.09	2.55	-	6.59	6.93	6.90	-
**11 Thr**	8.09	4.08	3.88	0.97	-	-	-	-
**12 Ser**	8.06	4.08	3.65	-	-	-	-	-
**13 Cys**	8.12	4.34	2.91 (β2)<br>2.73 (β3)	-	-	-	-	-

This analogue was highly insoluble and we could not determine its structure in solution.

Supplementary Note 2: Reverse phase – HPLC analysis of compounds

All analogues were dissolved in water 1 mg/mL and analysed by RP-HPLC.

RP-HPLC conditions:
Gradient: 5-85%B in 20 min, A: 0.01%TFA in H2O, B: 0.07% TFA in CAN
Column: Kromasil C8, 250 x 4.5, 5um
Wavelength: 220 nm; Flow: 1 mL/min, Tª: 25ºC
Equipment LC20 Shimadzu

Analogue 1 -RP - HPLC

Eluent A: H2O 0.1% TFA (RD_0605/5)
Eluent B: ACN 0.07% TFA (RD_0606/2)
Columna: Kromasil C8, 250x4.6, 5um, E159909
l:220nm; fluxe:1mL/min; Tª:25ºC
HPLC15

Chromatogram
A61 S1001 @D:\LabSolutions\Data\R+D\Analegs-SOM\SOM-610.lcd

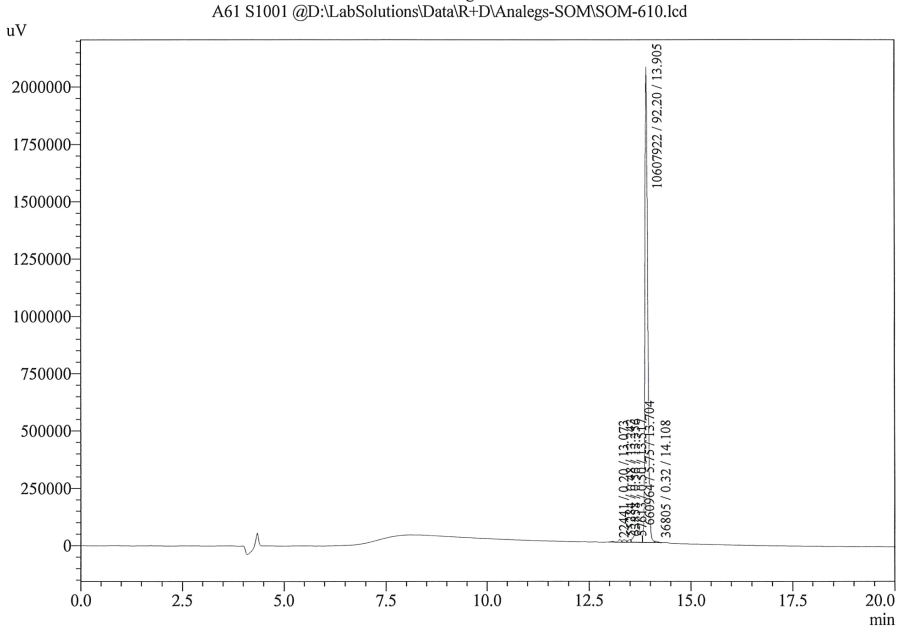

1 Det.A Ch1 / 220nm

PeakTable @D:\LabSolutions\Data\R+D\Analegs-SOM\SOM-610.lcd

Detector A Ch1 220nm

Peak#	Ret. Time	Area	Area %
1	13.07	22441	0.20
2	13.24	55284	0.48
3	13.36	63858	0.56
4	13.52	57613	0.50
5	13.70	660964	5.75
6	13.91	10607922	92.20
7	14.11	36805	0.32
Total		11504887	100.00

Analogue 2 -RP - HPLC

Eluent A: H2O 0.1% TFA (RD_0605/5)
Eluent B: ACN 0.07% TFA (RD_0606/2)
Columna: Kromasil C8, 250x4.6, 5um, E159909
l:220nm; fluxe:1mL/min; Tª:25ºC
HPLC15

Chromatogram
A63 15X1 @D:\LabSolutions\Data\R+D\Analegs-SOM\SOM-612.lcd

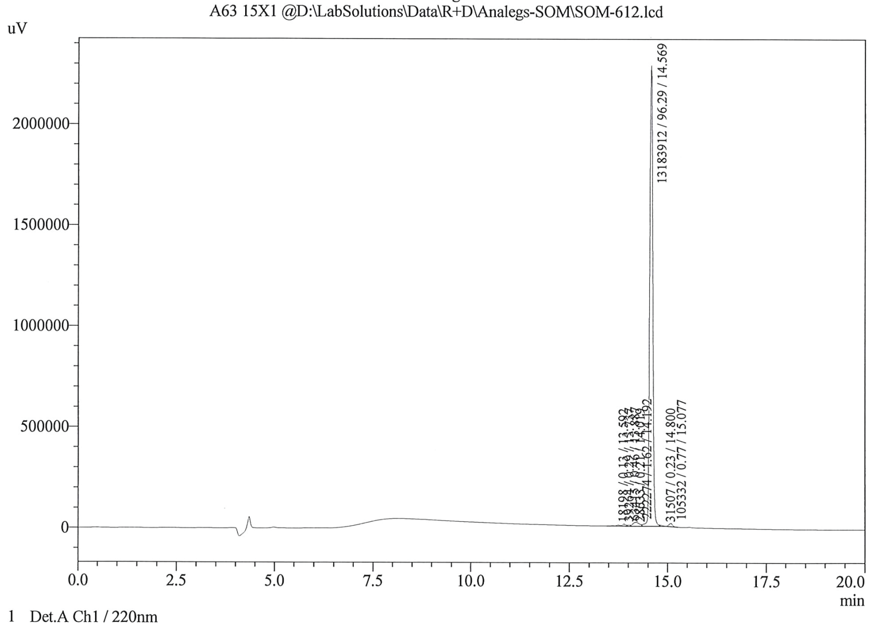

1 Det.A Ch1 / 220nm

PeakTable @D:\LabSolutions\Data\R+D\Analegs-SOM\SOM-612.lcd

Detector A Ch1 220nm

Peak#	Ret. Time	Area	Area %
1	13.59	18198	0.13
2	13.74	39264	0.29
3	13.89	63415	0.46
4	14.02	28633	0.21
5	14.19	222274	1.62
6	14.57	13183912	96.29
7	14.80	31507	0.23
8	15.08	105332	0.77
Total		13692535	100.00

Analogue 3 -RP - HPLC

Eluent A: H2O 0.1% TFA (RD_0605/5)
Eluent B: ACN 0.07% TFA (RD_0606/2)
Columna: Kromasil C8, 250x4.6, 5um, E159909
l:220nm; fluxe:1mL/min; Tª:25°C
HPLC15

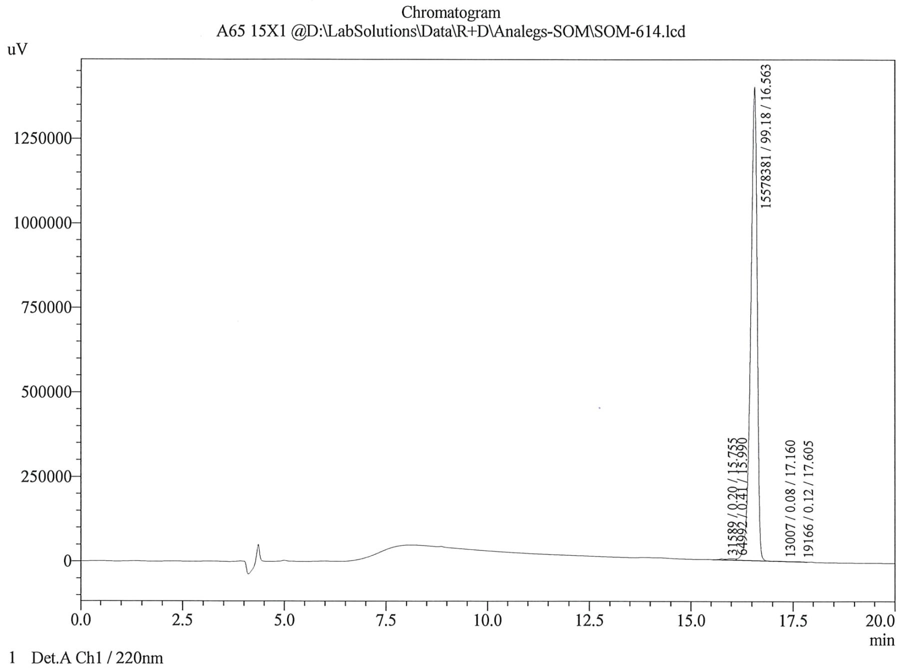

PeakTable @D:\LabSolutions\Data\R+D\Analegs-SOM\SOM-614.lcd

Detector A Ch1 220nm

Peak#	Ret. Time	Area	Area %
1	15.75	31589	0.20
2	15.99	64992	0.41
3	16.56	15578381	99.18
4	17.16	13007	0.08
5	17.60	19166	0.12
Total		15707134	100.00

Analogue 4 -RP - HPLC

Eluent A: H2O 0.1% TFA (RD_0605/5)
Eluent B: ACN 0.07% TFA (RD_0606/2)
Columna: Kromasil C8, 250x4.6, 5um, E159909
l:220nm; fluxe:1mL/min; Tª:25ºC
HPLC15

Chromatogram
A62 15X1 @D:\LabSolutions\Data\R+D\Analegs-SOM\SOM-611.lcd

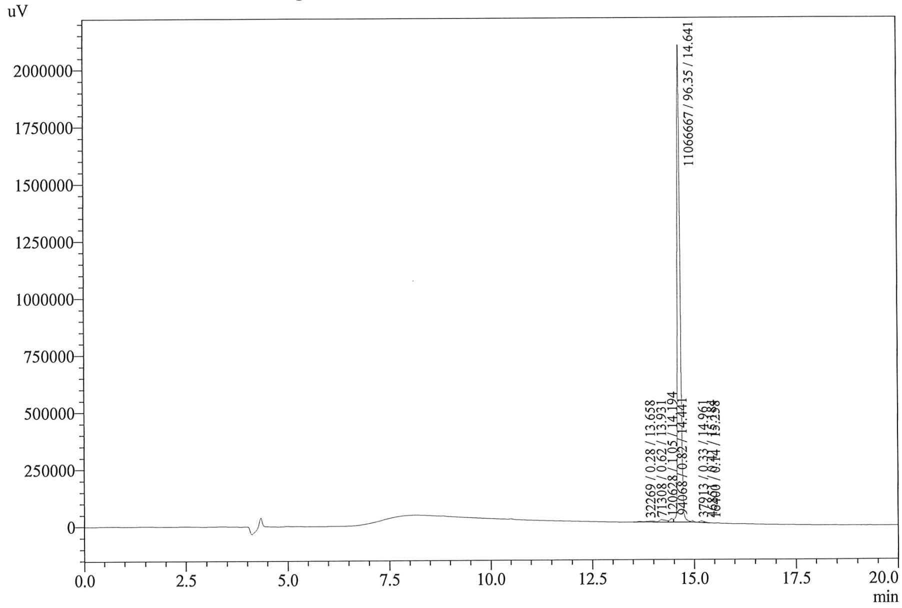

1 Det.A Ch1 / 220nm

PeakTable @D:\LabSolutions\Data\R+D\Analegs-SOM\SOM-611.lcd

Detector A Ch1 220nm

Peak#	Ret. Time	Area	Area %
1	13.66	32269	0.28
2	13.93	71308	0.62
3	14.19	120628	1.05
4	14.44	94068	0.82
5	14.64	11066667	96.35
6	14.96	37913	0.33
7	15.18	46851	0.41
8	15.26	16400	0.14
Total		11486103	100.00

Analogue 5 -RP - HPLC

Eluent A: H2O 0.1% TFA (RD_0605/5)
Eluent B: ACN 0.07% TFA (RD_0606/2)
Columna: Kromasil C8, 250x4.6, 5um, E159909
l:220nm; fluxe:1mL/min; Tª:25ºC
HPLC15

Chromatogram
A64 1401 reprocessat @D:\LabSolutions\Data\R+D\Analegs-SOM\SOM-613.lcd

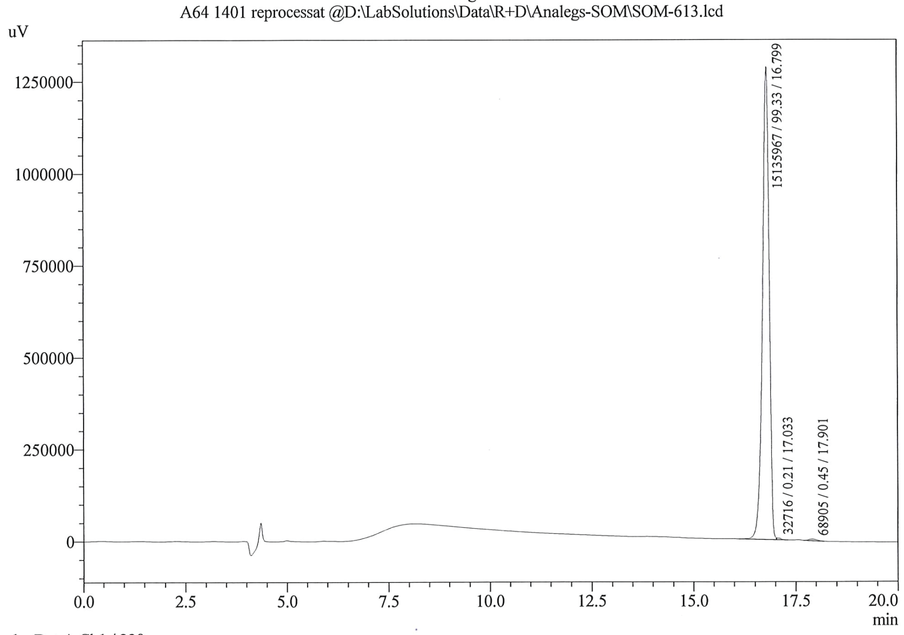

1 Det.A Ch1 / 220nm

PeakTable @D:\LabSolutions\Data\R+D\Analegs-SOM\SOM-613.lcd

Detector A Ch1 220nm

Peak#	Ret. Time	Area	Area %
1	16.80	15135967	99.33
2	17.03	32716	0.21
3	17.90	68905	0.45
Total		15237588	100.00

Analogue 6 -RP - HPLC

Eluent A: H2O 0.1% TFA (RD_0605/5)
Eluent B: ACN 0.07% TFA (RD_0606/2)
Columna: Kromasil C8, 250x4.6, 5um, E159909
l:220nm; fluxe:1mL/min; Tª:25ºC
HPLC15

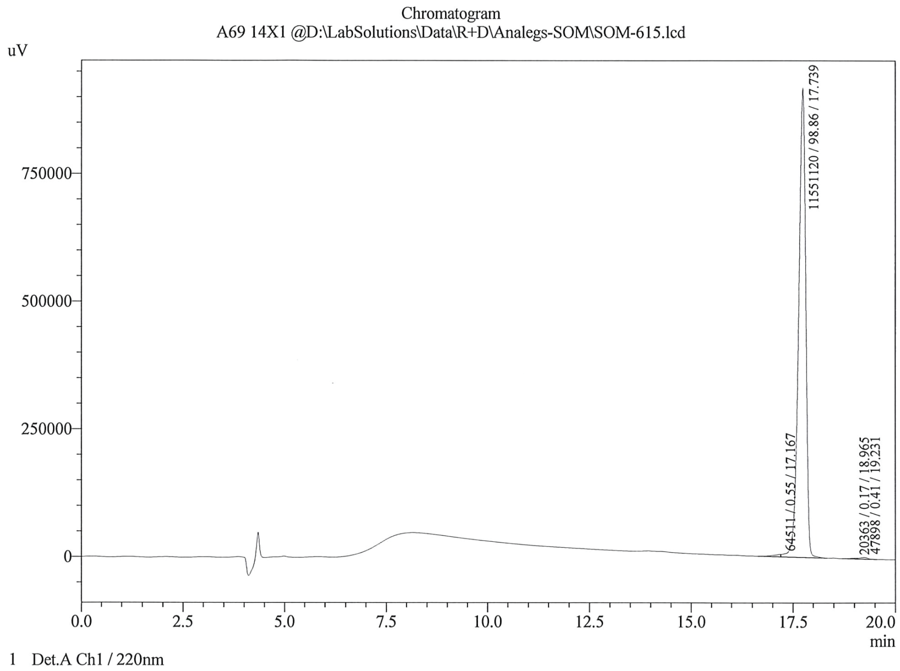

PeakTable @D:\LabSolutions\Data\R+D\Analegs-SOM\SOM-615.lcd

Detector A Ch1 220nm

Peak#	Ret. Time	Area	Area %
1	17.17	64511	0.55
2	17.74	11551120	98.86
3	18.97	20363	0.17
4	19.23	47898	0.41
Total		11683893	100.00

Supplementary Note 3: ESI-MS analysis of compounds

All analogues were dissolved in water 1 mg/mL and analyzed by Electrospray Ionization Mass Spectrometry.

ESI-MS conditions:
A: 0.01%TFA in H2O, B: 0.07% TFA in ACN
Equipment LCMS8040 Shimadzu

Analogue 1 -ESI-MS

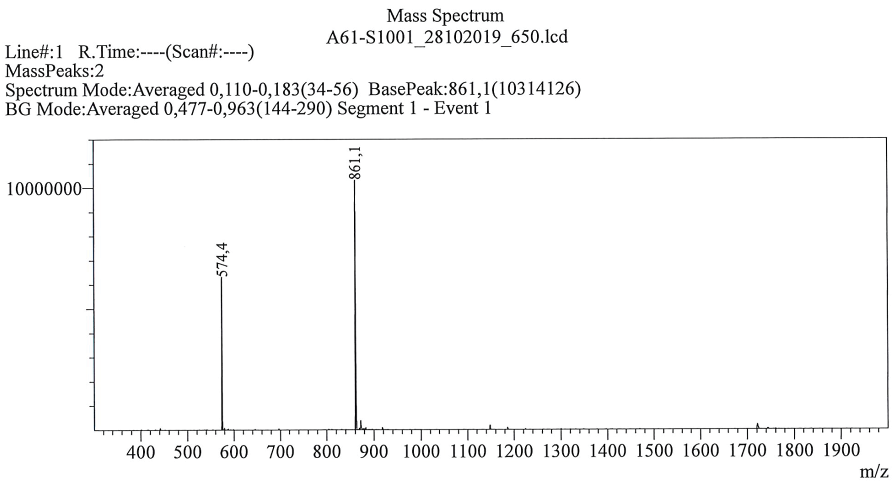

Analogue 2 -ESI-MS

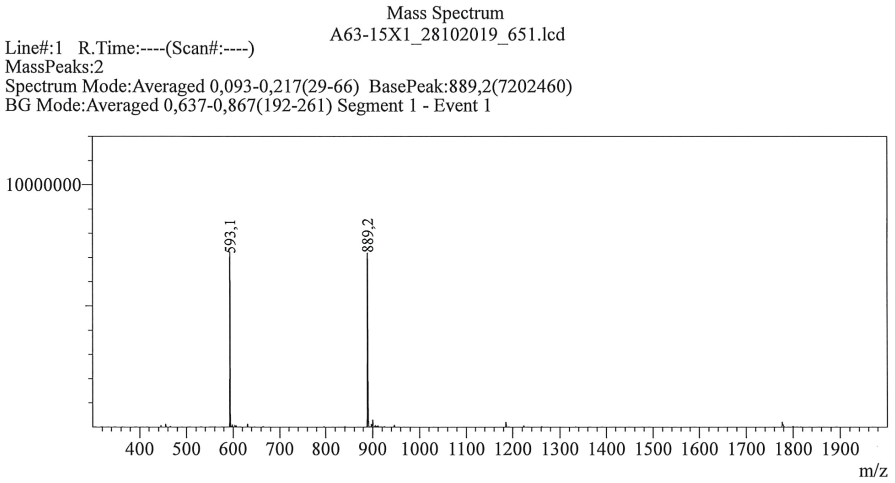

Analogue 3 -ESI-MS

Mass Spectrum
A65-15X1_28102019_653.lcd
Line#:1 R.Time:----(Scan#:----)
MassPeaks:2
Spectrum Mode:Averaged 0,100-0,207(31-63) BasePeak:952,3(8885292)
BG Mode:Averaged 0,557-0,827(168-249) Segment 1 - Event 1

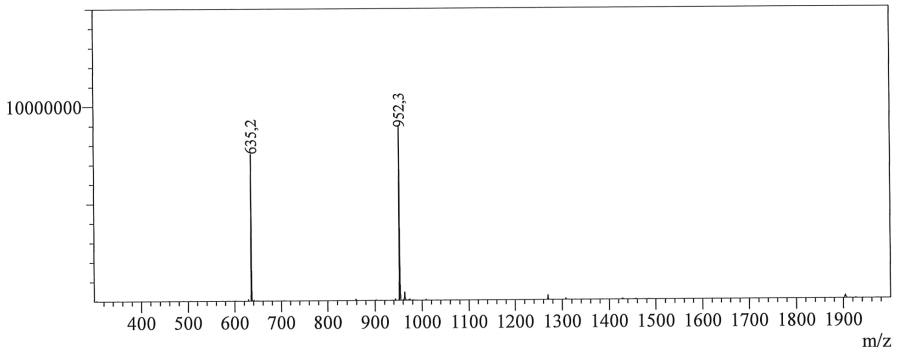

Analogue 4 -ESI-MS

Mass Spectrum
A62-15X1_28102019_649.lcd
Line#:1 R.Time:----(Scan#:----)
MassPeaks:2
Spectrum Mode:Averaged 0,093-0,207(29-63) BasePeak:889,1(7063200)
BG Mode:Averaged 0,427-0,913(129-275) Segment 1 - Event 1

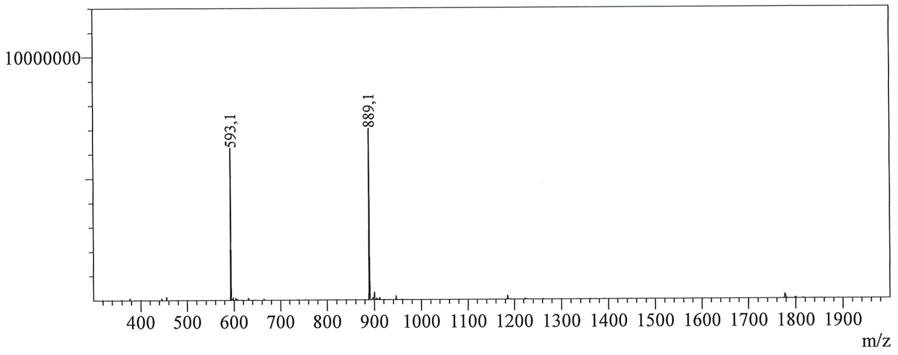

Analogue 5 -ESI-MS

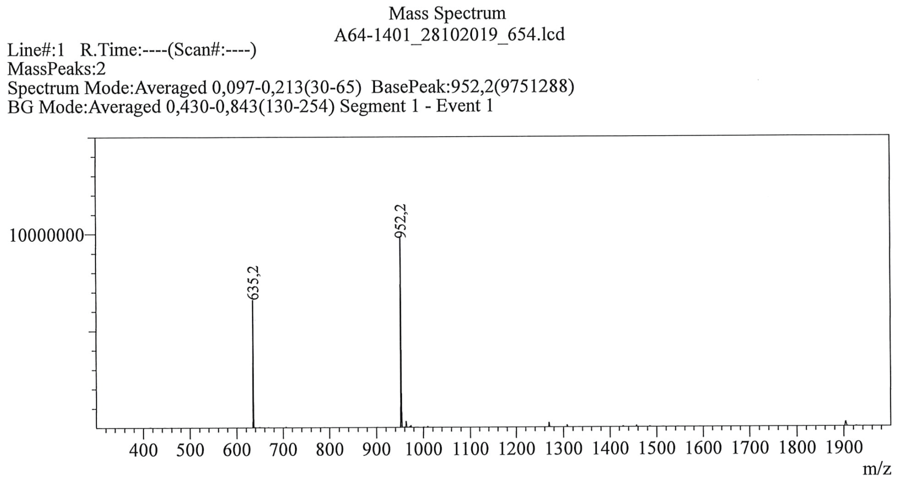

Analogue 6 -ESI-MS

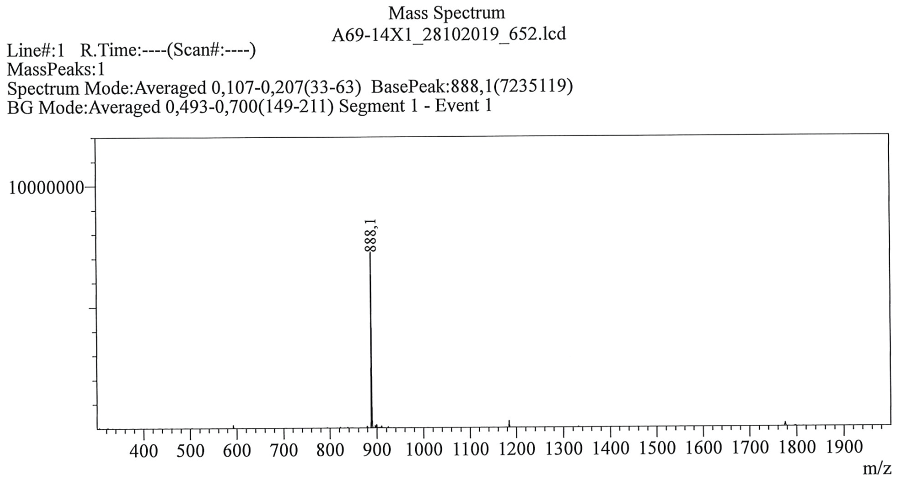

Supplementary References

1. Spier, A. D.; de Lecea, L. Cortistatin: a member of the somatostatin neuropeptide family with distinct physiological functions. *Brain Res Brain Res Rev* **2000,** *33* (2-3), 228-41.
