# Supplementary material for: Structure-based design of a Cortistatin analogue with immunomodulatory activity in models of inflammatory bowel disease
Source: Nat Commun. 2021 Mar 25;12:1869. doi: 10.1038/s41467-021-22076-5 (PMC7994712; doi:10.1038/s41467-021-22076-5)
Supplement: Supplementary file 3 — Reporting Summary [file 41467_2021_22076_MOESM3_ESM.pdf]

nature research

Corresponding author(s): Mario Delgado, Antoni Riera, and Maria J. Macias

Last updated by author(s): Jan 25, 2021

Reporting Summary

Nature Research wishes to improve the reproducibility of the work that we publish. This form provides structure for consistency and transparency in reporting. For further information on Nature Research policies, see Authors & Referees and the Editorial Policy Checklist .

Statistics

For all statistical analyses, confirm that the following items are present in the figure legend, table legend, main text, or Methods section.

n/a	Confirmed	
☐	☒	The exact sample size (*n*) for each experimental group/condition, given as a discrete number and unit of measurement
☐	☒	A statement on whether measurements were taken from distinct samples or whether the same sample was measured repeatedly
☐	☒	The statistical test(s) used AND whether they are one- or two-sided<br>*Only common tests should be described solely by name; describe more complex techniques in the Methods section.*
☒	☐	A description of all covariates tested
☒	☐	A description of any assumptions or corrections, such as tests of normality and adjustment for multiple comparisons
☐	☒	A full description of the statistical parameters including central tendency (e.g. means) or other basic estimates (e.g. regression coefficient) AND variation (e.g. standard deviation) or associated estimates of uncertainty (e.g. confidence intervals)
☐	☒	For null hypothesis testing, the test statistic (e.g. *F*, *t*, *r*) with confidence intervals, effect sizes, degrees of freedom and *P* value noted<br>*Give P values as exact values whenever suitable.*
☒	☐	For Bayesian analysis, information on the choice of priors and Markov chain Monte Carlo settings
☒	☐	For hierarchical and complex designs, identification of the appropriate level for tests and full reporting of outcomes
☒	☐	Estimates of effect sizes (e.g. Cohen's *d*, Pearson's *r*), indicating how they were calculated

Our web collection on statistics for biologists contains articles on many of the points above.

Software and code

Policy information about availability of computer code

Data collection	NMR data was collected using TopSpin 3.5, by Bruker BioSpin.
Data analysis	NMR data was analyzed using TopSpin 3.5, by Bruker BioSpin, CNS 1.2 (Brunger AT et al.) and CARA 1.4 (Rochus Keller)<br>GraphPad Prism 7.0 and 9.0 for statistical analysis.

For manuscripts utilizing custom algorithms or software that are central to the research but not yet described in published literature, software must be made available to editors/reviewers. We strongly encourage code deposition in a community repository (e.g. GitHub). See the Nature Research guidelines for submitting code & software for further information.

Data

Policy information about availability of data

All manuscripts must include a data availability statement. This statement should provide the following information, where applicable:

- Accession codes, unique identifiers, or web links for publicly available datasets
- A list of figures that have associated raw data
- A description of any restrictions on data availability

Cortistatin Analogue 5 structure is deposited into the PDB and BMRB databases with accession codes 6Y1Q and 34491 respectively. All relevant data are available from the authors upon request. Source data underlying Figs 2a-c, 3a–b, 4a-c, 5a-c and 6a-d, and Supplementary Figs 2a-d, 3, 4a-b, 5 and 6b-e are provided as a Source Data file.

Field-specific reporting

Please select the one below that is the best fit for your research. If you are not sure, read the appropriate sections before making your selection.

Life sciences study design

All studies must disclose on these points even when the disclosure is negative.

Sample size	Based in previous experience in these models, we estimated the size of each experimental group in approximately 8 mice, in order to reach a statistic detection power of 80% with a 30% of change, assuming a standard deviation of 30% and a level of significance <0.05% (GraphPad software)
Data exclusions	No data were excluded from the analysis.
Replication	All animal studies were performed in two independent experiments. In vitro experiments were replicated two (binding assay) and three (macrophage and spleen cell cultures) times. In all cases, replication of experiments was successful.
Randomization	Animals were randomly distributed in different experimental groups.
Blinding	Determination of body weight, colitis score, macroscopic colonic damage and histopathological analysis were determined in a blinded fashion by two independent researchers different than who made treatment.

Reporting for specific materials, systems and methods

We require information from authors about some types of materials, experimental systems and methods used in many studies. Here, indicate whether each material, system or method listed is relevant to your study. If you are not sure if a list item applies to your research, read the appropriate section before selecting a response.

Materials & experimental systems

n/a	Involved in the study	
☐	☒	Antibodies
☐	☒	Eukaryotic cell lines
☒	☐	Palaeontology
☐	☒	Animals and other organisms
☒	☐	Human research participants
☒	☐	Clinical data

Methods

n/a	Involved in the study	
☒	☐	ChIP-seq
☐	☒	Flow cytometry
☒	☐	MRI-based neuroimaging

Antibodies

Antibodies used	All antibodies used in this study are described in the methods section of the manuscript, including company supplier, specificity, labelling and clone.
Validation	All antibodies used in this study were validated by vendor companies: BD Bioscience (all the antibodies with the exception of anti-FoxP3) and eBiosecience (anti-FoxP3 Ab)

Eukaryotic cell lines

Policy information about cell lines

Cell line source(s)	Raw 264 mouse macrophagic cell line purchased from ATCC
Authentication	Provided by ATCC
Mycoplasma contamination	Raw 264 macrophagic cultures used in this study were negative in mycoplasm tests.
Commonly misidentified lines (See ICLAC register)	Does not apply to this study

Animals and other organisms

Policy information about studies involving animals; ARRIVE guidelines recommended for reporting animal research

Laboratory animals	Mouse: C57Bl/6 male 6-7 weeks-old and Balb/c male 6-8 weeks-old. Mice were housed in the SPF IPBLN-CSIC animal facility in a controlled-temperature/humidity environment (22 +/- 1°C, 60–70% relative humidity) in individual cages (8-10 mice per cage, with wood shaving bedding and nesting material), with a 12 h light/dark cycle (lights on at 0700 h) and were fed rodent chow (Global Diet 2018, Harlan) and tap water ad libitum. Mice were allowed to acclimatize to their housing environment for at least 5 days prior to experimentation and to the experimental room for 1 h before experiments. Animal studies are reported in compliance with the ARRIVE guidelines (Kilkenny et al., 2010; McGrath and Lilley, 2015).

Wild animals	This study did not involve wild animals
Field-collected samples	This study did not involve field-collected samples.
Ethics oversight	The experimental protocols of this study conform to EU Directive 2010/63 and followed the ethical guidelines for investigations with experimental animals approved by the Ethics Review Committee for Animal Experimentation of Spanish Council of Scientific Research.

Note that full information on the approval of the study protocol must also be provided in the manuscript.

Flow Cytometry

Plots

Confirm that:

- [x] The axis labels state the marker and fluorochrome used (e.g. CD4-FITC).
- [x] The axis scales are clearly visible. Include numbers along axes only for bottom left plot of group (a 'group' is an analysis of identical markers).
- [x] All plots are contour plots with outliers or pseudocolor plots.
- [x] A numerical value for number of cells or percentage (with statistics) is provided.

Methodology

Sample preparation	Single-cell preparations isolated from mouse mesenteric lymph nodes to analyze expression of CD25 and FoxP3 in CD4 T cells and lymph node cells activated with PMA+ConA in the presence of brefeldin to analyze intracellular cytokines in CD4 lymphocytes.
Instrument	FACScalibur flow cytometer, Becton Dickinson
Software	Acquisition with CellQuest software and analysis with CellQuest and FlowJo software.
Cell population abundance	This study did not require sorting cells.
Gating strategy	Gating strategy is described in detail in Supplementary Figure 8.

- [x] Tick this box to confirm that a figure exemplifying the gating strategy is provided in the Supplementary Information.
